# Acute Gastrointestinal Bleeding in Olmesartan-Associated Collagenous Gastroduodenitis: A Potential Endoscopic Complication

**DOI:** 10.1155/2018/3295204

**Published:** 2018-03-14

**Authors:** Rachel Hudacko, Lance Siegel

**Affiliations:** ^1^Orange Pathology Associates, PC, Department of Pathology, Orange Regional Medical Center, 707 East Main St., Middletown, NY 10940, USA; ^2^Horizon Family Medical Group, Department of Gastroenterology, Orange Regional Medical Center, 707 East Main St., Middletown, NY 10940, USA

## Abstract

Collagenous gastroenteritis is a rare disease that is known to be associated with the drug olmesartan, an angiotensin II receptor antagonist used to treat hypertension. It is characterized histologically by increased subepithelial collagen deposition with associated inflammation and epithelial injury. Endoscopically, the mucosa appears inflamed and friable and may be nodular or atrophic. We report a case of acute gastric bleeding on direct mucosal contact during endoscopy in a patient with olmesartan-associated collagenous gastroduodenitis to raise awareness of this potential endoscopic complication.

## 1. Introduction

Collagenous gastroenteritis is a pattern of injury that is characterized histologically by subepithelial collagen deposition >10 *μ*m in thickness with associated mucosal inflammation and epithelial injury [1]. The collagen bands are usually irregular, often entrap small capillaries, and can be highlighted with a trichrome stain. It is a rare disorder that may involve the stomach (collagenous gastritis) and/or small bowel (collagenous enteritis/sprue) and may occasionally coexist with the more common entity, collagenous colitis. The etiology of collagenous gastroenteritis is unknown but is suspected to be immune-mediated and may be associated with certain medications including the antihypertensive drug olmesartan, an angiotensin II receptor antagonist [2]. Several reports in the literature describe cases of olmesartan-associated collagenous duodenitis/sprue [3–5] with fewer reports of olmesartan-associated collagenous gastritis [3, 6] and only rare reports involving both the stomach and small bowel [3]. We herein report a case of olmesartan-associated collagenous gastroduodenitis that was complicated by acute gastrointestinal bleeding during endoscopy.

## 2. Case Presentation

A 64-year-old woman presented to the gastroenterologist with abdominal pain, unintentional 10 pound weight loss over 3 months, and reflux symptoms. Pertinent medical history included hypertension treated with olmesartan 20 mg once a day for the past 7 years, hyperlipidemia treated with simvastatin, gastroesophageal reflux disease treated with pantoprazole 20 mg twice a day, and osteoporosis treated with denosumab injections every 6 months. Esophagogastroduodenoscopy (EGD) performed in the office showed granular mucosa in the proximal duodenum and nodular mucosa in the stomach with numerous linear ulcers. On withdrawal of the scope from the duodenum, a large amount of fresh blood was noted in the stomach with clotted blood near the cardia. The bleeding was believed to be due to either the underlying gastric ulcers or a Mallory Weiss tear. The patient was transferred to the emergency department for further evaluation.

**Figure 1:**
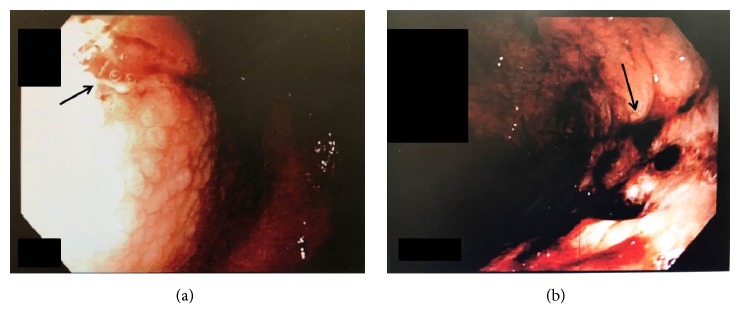
Endoscopic photos of the body of the stomach show diffusely nodular mucosa (a) with deep linear ulcers (arrows) and severe inflammation and hemorrhage (b).

**Figure 2:**
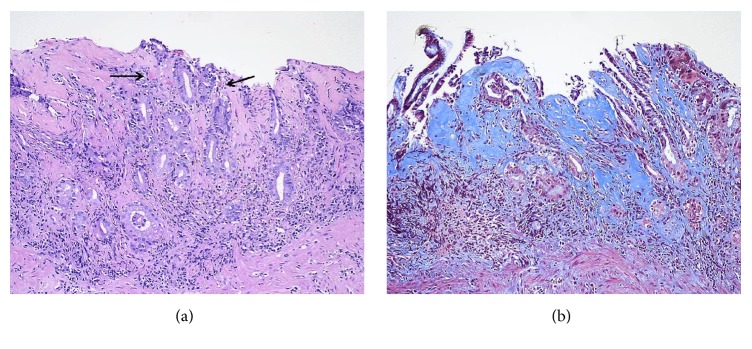
20x magnification: (a) hematoxylin and eosin-stained section of the gastric body shows a thick, markedly irregular pink band of subepithelial collagen deposition with complete surface erosion. The mucosa is inflamed and atrophic with loss of parietal cells. Arrows indicate entrapped capillaries. (b) Trichrome stain highlights the collagen blue.

**Figure 3:**
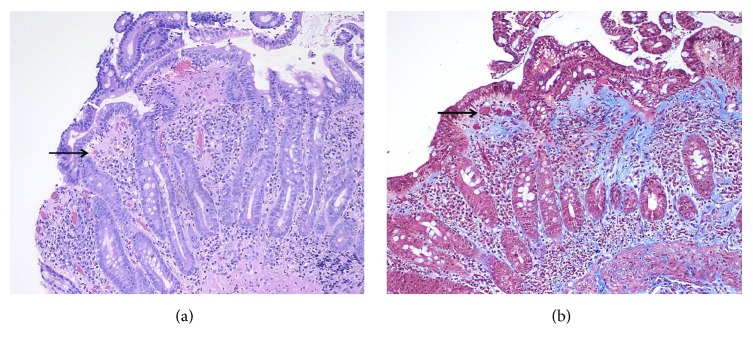
20x magnification: (a) hematoxylin and eosin-stained section of the duodenum shows villous shortening/partial atrophy with increased subepithelial collagen deposition (pink). (b) Trichrome stain highlights the collagen blue. Arrows indicate entrapped capillaries.

On admission, the hemoglobin and hematocrit were normal, and an emergent EGD was performed. The gastric fundus and body appeared atrophic with friable and severely inflamed mucosa and multiple linear ulcers without active bleeding (Figure 1). The antral mucosa appeared erythematous. Biopsies of the stomach and duodenum were performed. The biopsies of the antrum and body showed increased subepithelial collagen deposition confirmed on a trichrome stain, chronic inflammation in the lamina propria, surface epithelial regenerative changes, and erosions, consistent with collagenous gastritis (Figure 2). The gastric body mucosa was atrophic with near-total loss of parietal cells. Immunostain for* H. pylori* organisms was negative, and a Congo red stain for amyloid was negative. The biopsies of the duodenum showed partial villous atrophy and increased subepithelial collagen deposition confirmed on a trichrome stain, consistent with collagenous duodenitis/sprue (Figure 3). The patient was started on high dose acid suppression with pantoprazole 40 mg twice a day, and the olmesartan was discontinued.

**Figure 4:**
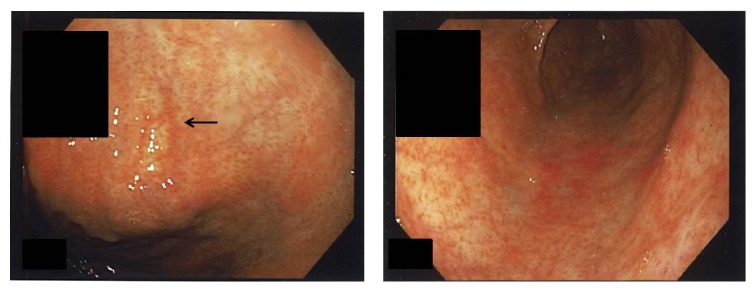
Endoscopic photos of the body of the stomach 7 weeks after cessation of olmesartan show atrophic mucosa with few residual areas of erythema (arrow) and complete resolution of the ulcers.

Follow-up EGD 7 weeks later showed somewhat atrophic gastric mucosa with few residual linear areas of erythema and complete resolution of the ulcers, inflammation, and mucosal friability (Figure 4). The duodenal mucosa appeared normal. Colonoscopy was unremarkable. Repeat biopsies showed marked improvement of the collagenous gastritis with resolution of the epithelial injury and only rare residual foci of subepithelial collagen. A mild nonspecific chronic inactive gastritis remained present. The biopsies from the duodenum and left colon showed normal mucosa without evidence of collagenous duodenitis or collagenous colitis.

## 3. Brief Discussion

Olmesartan-associated collagenous gastroenteritis is a rare entity that may be immune-mediated in nature. The length of time of olmesartan use before onset of symptoms varies from less than 1 month to 11 years [7]. When the stomach is solely involved, symptoms include dyspepsia, anemia due to bleeding, weight loss, and diarrhea. When the small intestine is involved, the most common symptoms are watery diarrhea and weight loss due to malabsorption [2]. The characteristic endoscopic finding of collagenous gastritis is nodular mucosa. Other findings include erythema, mucosal friability, erosions, ulcers, and atrophy [2, 6]. The endoscopic findings in the small bowel are nonspecific and include pale mucosa, mucosal thickening, and scalloping [2, 4].

The diagnostic histologic feature of collagenous gastroenteritis is band-like layer of subepithelial collagen deposition that is at least 10 *μ*m in thickness and is associated with a chronic inflammatory infiltrate and surface epithelial injury [1]. The deposits can be patchy and irregular and often entrap superficial dilated capillaries [2]. Bleeding results from surface epithelial injury and sloughing which exposes these capillaries.

The majority of cases of olmesartan-associated collagenous gastroenteritis reported in the literature resolved after cessation of the drug [7]. One study reported complete clinical and pathologic resolution of collagenous duodenitis after treatment with the immunosuppressive drugs FK-506 and rapamycin [8]. Our patient had a complete clinical response and near-complete pathologic response at 7-week follow-up after discontinuation of olmesartan.

In summary, we report a case of acute gastric bleeding during endoscopy due to olmesartan-associated collagenous gastroduodenitis. The mechanism of bleeding in this disease is the presence of abnormal subepithelial collagen causing mucosal fragility. Bleeding may be spontaneous and chronic with associated anemia or may be acute and caused by direct contact with instruments during endoscopy, as was believed to be the reason for the acute bleed in this case. Although rare, it is important for endoscopists to be aware of this potential endoscopic complication in patients taking olmesartan.
